# Screening for atrial fibrillation in older adults in primary care: a cross-sectional pilot study in Qatar

**DOI:** 10.3389/fpubh.2026.1788553

**Published:** 2026-07-17

**Authors:** Ahmed Sameer Alnuaimi, Shahid Ahmad, Abdullah Syed, Babur Khan, Zaheer Ahmed, Tahir Hameed, Muslim Abbas Syed, Mohamed Ahmed Syed

**Affiliations:** 1 Primary Health Care Corporation, Doha, Qatar; 2 Hamad Medical Corporation, Doha, Qatar

**Keywords:** atrial fibrillation, older adults, primary care, public health, Qatar, screening, stroke prevention

## Abstract

**Background:**

Atrial fibrillation (AF) is the most common arrhythmia in clinical practice, and its prevalence is increasing in aging populations. It is a leading cause of acute stroke, heart failure, and other cardiovascular morbidities. AF is often asymptomatic and is frequently diagnosed late, typically after a patient presents with acute stroke. Early detection and treatment can prevent such life-threatening events, making AF screening a public health priority. However, diagnosing asymptomatic AF remains challenging. While international guidelines recommend AF screening, Qatar lacks documented prevalence data and formal screening programs. This study aimed to assess the feasibility of an AF screening program for older adults in Qatar.

**Methods:**

A cross-sectional study was conducted among individuals aged ≥65 years registered with the Primary Health Care Corporation (PHCC)-Qatar, which serves approximately 70% of Qatar's population. A simple random sample of 139 to 385 individuals was determined to ensure a sampling error of 5% to 3%, assuming a 10% AF prevalence. The participants underwent screening via standard ECG tracing that were reviewed independently by both the family physicians and a cardiologist.

**Results:**

The prevalence of AF among the study population was 5.4%. Newly diagnosed cases accounted for 2% of the 249 participants screened. All newly detected patients had CHA_2_DS_2_-VA scores indicating the need for anticoagulant therapy to reduce stroke risk. Furthermore, compared with cardiologists, the family physicians in primary care successfully identified 75% of AF cases through ECG.

**Conclusions:**

If implemented nationally among individuals aged ≥65 years, the screening program could identify 747 undiagnosed AF cases among the 37,371. AF screening for individuals aged ≥75 years appears to provide an effective strategy for reducing the cost and supporting the implementation of a targeted screening strategy in Qatar.

## 1 Introduction

Atrial fibrillation (AF) is a cardiac arrhythmia characterized by supraventricular tachyarrhythmia with uncoordinated atrial electrical activation, leading to ineffective atrial contraction (1). It is the most common arrhythmia encountered in clinical practice, affecting approximately 59.7 million people worldwide (2). Aging is the most significant risk factor for AF. By 55 years of age, the lifetime risk of developing AF is 37% (3). Additionally, males are nearly twice as likely as females to develop this condition (4).

AF is a major contributor to stroke, heart failure, and cardiovascular morbidity (2). It is associated with a fivefold increase in stroke risk, a threefold increase in heart failure risk, and a twofold increase in the risk of dementia and mortality (5). Stroke remains a significant public health challenge worldwide, contributing substantially to morbidity and mortality (6). AF is implicated in approximately 20–30% of ischemic strokes and 10% of hemorrhagic strokes (1).

Clinically, AF is often asymptomatic and is frequently diagnosed only when patients present with acute stroke (7). Importantly, the risk of ischemic stroke in AF patients is comparable regardless of whether symptoms are present (8). Early detection of AF is crucial, as timely management can significantly reduce associated risks. For patients with AF, oral anticoagulation (OAC) therapy can lower stroke risk by up to two-thirds, underscoring the importance of early diagnosis and intervention.

Despite the benefits of early detection, asymptomatic AF remains a diagnostic challenge. International clinical guidelines and expert consensus strongly advocate for AF screening (1, 9, 10). However, the optimal methods, locations, and target populations for screening should align with the specific needs of a healthcare system. European guidelines (ESC) recommend opportunistic AF screening in individuals aged ≥65 years and suggest systematic ECG-based screening in those aged ≥75 years or at high stroke risk (11). In contrast, U.S. recommendations are more cautious: the USPSTF concludes that evidence is insufficient to support routine ECG-based screening for AF in asymptomatic adults, and contemporary ACC/AHA/HRS guidelines acknowledge incident and device-detected AF but do not endorse population-wide systematic screening programs (12).

In Qatar, AF was identified in 6.4% of patients who experienced a stroke (13). Nevertheless, the true prevalence of AF in the general population has not been documented, and there are currently no formal AF screening programs. This study aims to assess the feasibility of an AF screening program targeting individuals aged 65 and older in Qatar's primary care setting via a 12-lead electrocardiogram (ECG).

## 2 Methods

### 2.1 Study setting

The study was undertaken in Qatar's publicly funded primary care organization, the Primary Health Care Corporation (PHCC). It has 31 primary health care centers across three geographical regions (central, northern and western) of the country. Approximately 70% of Qatar's population is registered with the PHCC.

### 2.2 Study population and data collection

The study population consisted of individuals aged ≥65 years who were registered at a PHCC health center. Since participants were to be contacted by phone, the sampling frame was limited to those with an available phone number, representing more than 95% of the reference population. On the basis of literature, an AF prevalence rate of 10% was assumed. To achieve a 95% confidence level with a margin of error ranging from 5% to 3%, a sample size of 139 to 385 participants was deemed sufficient. This sample size was considered more than adequate for a pilot study.

A list of all individuals meeting the inclusion criteria was extracted from the PHCC's electronic medical records. The population sampling frame consisted of 37,371 with a valid phone number out of the total 38,049 eligible population. Contact info missing data constituted < 2%. Previous experience indicated a low response rate to healthcare surveys of < 10% in PHCC. Four lists of 1,000 randomly selected sampling units (individuals) were planned to be administered consecutively by six data collector teams operating in 6 health centers to achieve a maximum sample size of 385 during the allocated period of 6 weeks. The teams managed to administer three of the sampling lists (3000) with a response rate of 8.3%, resulting in a sample size of 249 during the allocated time for data collection. A nurse contacted potential participants by phone to inform them about the study. Those who agreed to participate were scheduled for an appointment at one of six designated PHCC health centers.

At the appointment, a nurse obtained informed consent and collected demographic and clinical information, including age, sex, height, weight, blood pressure, and medical history. A 12-lead ECG was performed to screen for AF. The ECG results were reviewed independently by a family medicine physician and a cardiologist. In addition, the data required for calculating the CHA_2_DS_2_-VA score (risk factors for AF) were recorded. If AF was confirmed, the participant was scheduled for a detailed follow-up appointment with a family physician. During this appointment, the diagnosis was explained, additional investigations were ordered as needed, and a referral to a cardiologist was arranged, Appendix 1.

### 2.3 Data analysis

All the data were analyzed via the IBM Statistical Package for the Social Sciences (IBM SPSS) software V28. Basic descriptive statistics were used to describe the ECG results, diagnoses made by family physicians and cardiologists, and associated risk factors. Odds ratios were calculated to assess the strength of the associations between AF and various risk factors.

### 2.4 Ethics approval and consent to participate

The study presented a minimal risk of harm to the participants. The data collected was anonymized. None of the subjects' personal information was available to the research team. An informed signed consent was secured from the study participants to assure their voluntary participation. Overall, the study was conducted with integrity according to generally accepted ethical principles in Belmont report and Helsinki's declaration. The study protocol was reviewed and approved by the PHCC's independent ethics committee (PHCC/DCR//2020/01/005).

## 3 Results

A total of 249 individuals participated in the study. Overall, the ECG screening identified 7 individuals (2.8%) diagnosed with AF, 5 of them (2%) were new cases with no previous history of AF, while the other 2 (0.8%) are already known to have AF (recorded in the EHR). Another 95 individuals (38.1%) had other diagnoses of EGC; 90 (36.1%) of them had no previous history of AF, and 5 (2%) had a previous history of AF (see Table 1). In conclusion, the prevalence rate of AF, whether newly diagnosed or previously recorded in the EHR was 6% (95% confidence interval of 3–9%) in the screened cohort. The electronic health records (EHR) of PHCC-registered adults aged 65 years and older with valid phone numbers were reviewed for an existing diagnosis of AF. The prevalence rate of AF in the reference population was 5.4% (2,025 out of 37,371 individuals). On the basis of the results of the current study (2% probability for the screening program to identify new AF cases among individuals in this age group), it is anticipated that 747 previously undiagnosed AF cases could be detected. The number of undiagnosed cases is projected to range from 112 (at the most conservative end of the 95% confidence interval) to 1,420 (at the upper limit of the interval).

**Table 1:** Frequency distribution of the final diagnoses by cardiologist assessment of current ECGs and past history of AF in the electronic health records.

Final diagnosis	*N*	%	95% Confidence Interval (%)
No abnormal ECG findings (including AF)	144	57.8	
Newly diagnosed (current ECG)	5	2.0	0.3–3.8
Past history of AF confirmed by current ECG	2	0.8	0.02–0.29
Past history of AF with negative current ECG	3	1.2	0.4–3.5
Past history of AF presenting with other ECG findings apart of AF	5	2.0	0.3–3.8
Other positive ECG findings	90	36.1	30.2–42.1
Total	249	100.0	

As shown in Table 2, the family physician at the primary health center demonstrated a sensitivity of 71.4% in detecting AF through ECG compared with the gold-standard diagnosis by a cardiologist evaluating the same ECG. The specificity was nearly perfect at 99.2%. Given the very low prevalence of newly diagnosed AF in the study (pretest probability of only 2.8%), a positive AF diagnosis made by the family physician via ECG had a positive predictive value (PPV) of 71.4%. On the other hand, a negative ECG result from the same physician excluded an AF diagnosis with very high confidence, as reflected by a negative predictive value (NPV) of 99.2%.

**Table 2:** ECG findings of the family medicine physician compared with those of a cardiologist.

Family physician ECG findings	Cardiologist ECG review			
	Normal	AF	Other positive findings	Total
Normal	136	0	32	168
AF	1	5	1	7
Other positive findings	10	2	62	74
Total	147	7	95	249

AF diagnosis by the family physician is associated with the following test performance characteristics: Sensitivity = 71.4%, Specificity = 99.2%, PPV = 71.4%, NPV = 99.2%, Pretest probability = 2.8%, Accuracy = 98.1.

Similarly, as shown in Table 3, the most commonly reported risk factor in the study sample was high blood pressure (62.7%), followed by diabetes mellitus (57%) and female sex (45%). The participants aged 75 years and older constituted 28.5% of the sample. Vascular disease, including angina or myocardial infarction, was reported in 9.2% of individuals, whereas a history of stroke or transient ischemic attack was noted in 5.6%. The least frequently reported risk factor was congestive heart failure, which was present in only 1.2% of the sample.

**Table 3:** The prevalence rates of selected risk factors.

Positive risk factors for AF (*N* = 249)	*N*	%	95% Confidence Interval (%)
High blood pressure	156	62.7	56.6–68.7
Diabetes mellitus	142	57.0	50.9–63.2
Female sex	112	45.0	38.8–51.2
Age 75+	71	28.5	22.9–34.1
Vascular disease (Angina, myocardial infarction)	23	9.2	5.6–12.8
Previous stroke or Transient ischemic attack (TIA)	14	5.6	2.8–8.5
Congestive heart failure	3	1.2	0.4–3.5

Table 4 shows that the strength of the associations between the seven risk factors and a positive diagnosis of AF, when tested independently, varied widely. There was no association observed for previous stroke or sex, whereas congestive heart failure was associated with up to a 20-fold increase in risk. However, owing to the rarity of AF, only the age criterion (being older than 74 years) demonstrated a statistically significant association, increasing the risk of having AF by 6.7 times.

**Table 4:** Risk of having AF according to selected risk factors.

Explanatory variable tested		Confirmed atrial fibrillation (Cardiologist)								
		Negative		Positive		Total	
		*N*	%	*N*	%	*N*	%	OR	95% CI (OR)	*P*
1.	Congestive heart failure Negative Positive	240 2	97.6 66.7	6 1	2.4 33.	246 3	100.0 100.0	20	(1.6–251.9)	0.08[NS]
2.	Age group 65–74 75+	176 66	98.9 93.0	2 5	1.1 7.0	178 71	100.0 100.0	6.7	(1.3–35.2)	0.021
3.	Vascular disease (Angina, myocardial infarction) Negative Positive	221 21	97.8 91.3	5 2	2.2 8.7	226 23	100.0 100.0	4.2	(0.8–23)	0.13[NS]
4.	High blood pressure Negative Positive	92 150	98.9 96.2	1 6	1.1 3.8	93 156	100.0 100.0	3.7	(0.4–31.1)	0.26[NS]
5.	Diabetes mellitus Negative Positive	105 137	98.1 96.5	2 5	1.9 3.5	107 142	100.0 100.0	1.9	(0.4–10.1)	0.48[NS]
6.	Previous stroke or transient ischemic attack (TIA) Negative Positive	228 14	97.0 100.0	7 0	3.0 0.0	235 14	100.0 100.0	^**^	^**^	1[NS]
7.	Sex Female Male	109 133	97.3 97.1	3 4	2.7 2.9	112 137	100.1 100.0	1[NS]	1.1	(0.2–5)

^**^Can not be calculated

## 4 Discussion

This cross-sectional study is exceptional in being the first conducted in Qatar and the Middle East to explore the risk of AF in the general population. Although extensive clinical research on this topic has been carried out in secondary and tertiary care settings in the region, no studies have focused on the general population.

While designed as a pilot study with a relatively small sample size and relying on a single ECG measurement, the use of robust random sampling allowed for meaningful preliminary prevalence estimates and identified potential gaps in the management of missed cases in primary care.

The current study demonstrated that systematic screening via ECG can identify new (previously undiagnosed) cases of AF in 2% of screened individuals aged 65 years and older. All newly detected patients had CHA_2_DS_2_-VA scores ranging from 3 to 5, indicating that they were eligible for anticoagulant therapy (14). EHR-based case identification, which is based on a history of AF recorded in electronic health records, revealed that 10 individuals (4%) had been previously labeled as AF cases. However, only 2 of these individuals (0.8%) were confirmed to have AF on the new screening ECG. This discrepancy may be attributed to paroxysmal AF being missed by a single-point ECG and highlights the need for prolonged rhythm monitoring to increase the sensitivity of AF detection. Using handheld ECG devices for 2 weeks or Holter patch monitoring for 5–7 days could significantly improve the sensitivity of detecting AF cases (14). It's worth noting here that there is an ongoing debate about the role of screening for AF in preventing/reducing future cardiovascular accidents (stroke in specific). Findings from the STROKESTOP I clinical trial suggest that atrial fibrillation screening provides a modest net benefit compared with standard care and can be safely implemented in older populations (15). The LOOP Study, a randomized controlled trial, showed that screening with an implantable loop recorder (ILR) tripled the detection of atrial fibrillation and increased anticoagulation initiation, yet it did not lead to a significant reduction in stroke or systemic arterial embolism (16). The evidence clearly shows that, in patients with atrial fibrillation treatment with oral anticoagulants reduces stroke incidence by 64% and mortality by 26%. However, uncertainty remains about whether atrial fibrillation detected through screening is equally beneficial in reducing morbidity and mortality carries, especially when screening is performed more intensively than a single time-point assessment (17).

The overall prevalence of AF in this study was 6% among those older than 65 years, which is approximately half of the prevalence reported in Western populations, such as the United States (13.2%) (18). This disparity may be attributed to the demographic composition of the study population, which consisted predominantly of Asian and, to a lesser extent, African individuals. AF incidence and prevalence are reportedly lower among Asians and black individuals than among those of European ancestry (19).

The adoption of an AF screening program is projected to identify approximately 747 previously undiagnosed AF cases among the 37,371 individuals aged 65 years and older within the PHCC-served population. This number is anticipated to increase in the future due to the increasing life expectancy of the population.

The criteria for CHA_2_DS_2_-VA scoring, excluding female sex, which is used to evaluate the eligibility of AF patients for anticoagulation therapy, are also established risk factors for developing AF (19). In the present study, diabetes and hypertension were present in more than half of the participants, whereas vascular disease and a history of stroke were reported in fewer than 10% of them. The strongest association with AF, however, was observed in individuals with congestive heart failure and those aged 75 years or older.

Congestive heart failure increased the risk of AF by 20-fold, although this association did not achieve statistical significance due to the low prevalence of heart failure in the study population. Conversely, being older than 74 years significantly increased the risk of AF by 6.7 times compared with younger individuals. Only 1.1% of participants under 75 years of age presented with AF on ECG, whereas 7% of those aged 75 years or older presented with AF. Similar findings were reported in a secondary analysis of the VITAL-AF trial conducted in the United States (18). In this context, one should highlight the fact that these associations and risk estimates were underpowered in the current study as reflect in the wide confidence interval for the calculated odds ratio.

Age would be a critical consideration in implementing a screening program in Qatar, as the older population is relatively small, thereby substantially reducing the cost of such a program. A large population-based study in China emphasized aging as the most significant risk factor for AF, alongside obesity, heart failure, vascular disease, hypertension, and diabetes. This study also highlighted a critical gap in preventing AF-related morbidity, as only 4% of eligible patients were prescribed anticoagulation therapy, despite a median CHA_2_DS_2_-VA score of 2 in the population (20).

One key outcome of this study was the demonstrated ability of family physicians (FPs) in primary care, as frontline healthcare providers, to detect approximately three-quarters of AF cases through ECG interpretation compared with cardiologists. Moreover, FPs exhibited near-perfect specificity in excluding a possible diagnosis of AF. However, the sensitivity observed in this study may be subject to instability due to statistical bias arising from the small number of positive AF cases (only seven) identified. The overall diagnostic accuracy of screening ECGs for AF by family physicians was 98.1%, which aligns with findings reported in the literature (21). Proposed strategies to increase the effectiveness of AF screening programs include fostering collaboration between FPs and cardiologists for ECG interpretation (22), providing targeted training to improve diagnostic performance, or incorporating artificial intelligence (AI) technologies to support ECG assessment. ECG analyses using AI engine have been shown to improve diagnosis and management. The AI diagnostic performance is comparable to that of experienced cardiologists (23). One plausible candidate for this purpose is PMcardio (24). These measures could significantly enhance the outcomes of a future AF screening program if implemented.

### 4.1 Study limitations

The study recruitment procedures relying on telephonic contact resulted in a low response rate of 8.3%. This limitation merits a separate study. It pointed out that success of systematic screening in older populations is often tied to broader digital access and community-based health interventions (25). In addition, future national programs might benefit from implementing a social health marketing mix to influence health-seeking behavior and improve case detection (26).

The calculated 2% prevalence rate of newly detected AF cases in this pilot study is subject to the wide margin of error limitation, ranging from 0.3% to 3.8%. However, this limitation does not diminish its value in informing the decision to implement a screening program, particularly as the population of older adults in Qatar continues to grow. Furthermore, targeting individuals aged 75 years and older lowers costs, supporting targeted screening currently. It is important to acknowledge that relying on a single ECG measurement to screen for AI may have underestimated the real prevalence of AF and reduced the sensitivity of screening. The effect of this underestimation is not expected to affect future morbidity and mortality as was suggested by the UK National Screening Committee and the US Preventive Services Task Force (USPSTF) recommendations (17). Moreover, this study was designed as a pilot to assess feasibility rather than to provide precise national estimates of undiagnosed atrial fibrillation. Consequently, projections of national case detection are based on a small number of newly identified cases and are subject to uncertainty, as reflected by the wide confidence intervals around the observed screening yield. The findings should therefore be interpreted as indicative of potential scale and resource implications, emphasizing the need for larger implementation studies that prospectively evaluate screening yield, participation rates, and clinical workload. Furthermore, while the study was not designed to include a formal economic evaluation, the findings identify clear priorities for future research to inform evidence-based policy decisions on atrial fibrillation (AF) screening in primary care. Future studies could incorporate a micro-costing or activity-based costing approach to quantify the personnel and operational resources required for screening, including nurse-led patient contact, ECG acquisition, and ECG interpretation by primary care physicians with specialist support as needed (27–29). Such analyses would allow estimation of the cost per AF case detected, a pragmatic metric frequently used by policy makers when assessing screening feasibility (30). In line with European Society of Cardiology (ESC) guidance, a budget impact analysis comparing opportunistic screening in individuals aged ≥65 years with targeted systematic screening of those aged ≥75 years would be particularly relevant, given the substantially higher detection yield observed in the older age group in this study (31, 32). To inform longer-term policy planning, future research may apply decision-analytic modeling (e.g., decision tree or Markov models) to project downstream outcomes such as anticoagulation initiation, stroke events averted, and healthcare utilization under alternative screening strategies (33, 34). Collectively, these approaches would support proportionate, age-targeted AF screening policies by providing structured evidence on resource requirements and potential value for money, consistent with contemporary European screening frameworks.

In conclusion, this pilot study highlights the feasibility and potential benefits of systematic AF screening in Qatar's primary care setting, particularly among individuals aged 75 years and older. Despite the limitations of the small sample size and wide margin of error, the findings favored targeted screening to detect undiagnosed AF patients, enabling timely intervention and reducing associated morbidity. Collaborative efforts, enhanced training, alternative screening strategies like intermittent or continuous ECG-based devices, and AI integration could further improve screening accuracy and may be considered for future research projects.

## Figures and Tables

**Figure A1:**
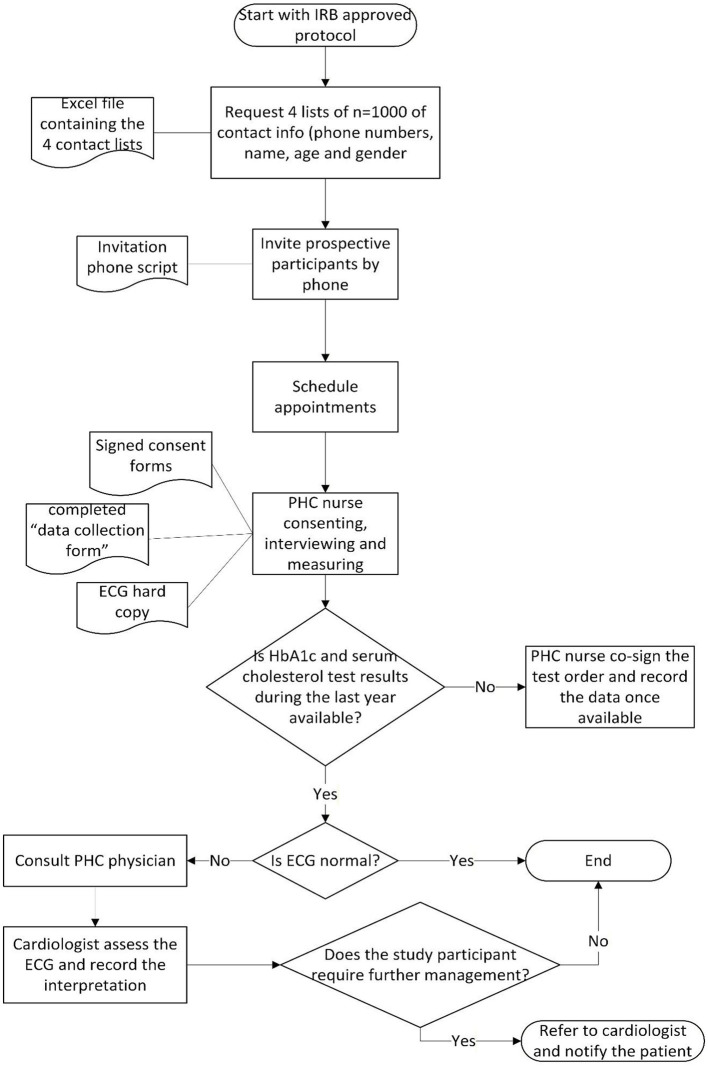
Flow diagram illustrating the data collection workflow.

## Data Availability

Restrictions apply to the datasets: The datasets presented in this article are not readily available because the Primary Health Care Corporation, Qatar, has a data acquisition and sharing policy that prohibits data release without an agreement between the relevant parties. Requests to access the datasets should be directed to PHCC, Qatar by email at researchsection@phcc.gov.qa.
